# 

**Published:** 1917-11-24

**Authors:** 


Rates of Subscription: Twelve months, 6/6; Six months, 4/-; three months, 2/-, post free to any address in the United Kingdom. Abroad, Twelve months, 8/8; Six months, 5/-; Three months, 2/6, post free. THE HOSPITAL "Index" to Volume LXII., April 1917 to September 1917, is now ready, price 2½d. per copy, post free. Address The Manager, The Hospital, "The Hospital" Building, 28/29 Southampton Street, Strand, London, W.C. 2.

### "All About the United States Navy."

A USEFUL little book under the above title has been published by Messrs. Sampson Low, Marston and Co., Ltd., at the price of 1s. net. Excellent illustrations of each type of vessel are given, and there is much information in a concise form respecting the sea forces of our new Allies.

### Personalia.

The Lincoln City Council on November 6 appointed Dr. C. W. Dias, of the City Fever Hospital, Lodge Moor, Sheffield, as assistant medical officer of Lincoln and assistant schools medical officer, at a salary of £350 a year, in the place of Dr. Ramsbottom, who has been called up for military service.

Alderman Dr. John Utting, who has taken a prominent part in connection with the health and sanitation of the city and port of Liverpool ever since he was elected a member of the City Council in 1889, was elected the new Lord Mayor of Liverpool on November 9. He has practised in the city for many years. At present he holds the rank of major in the R.A.M.C.(T.).

